# Prevalence of Adult Honey Bee (*Apis mellifera* L.) Pests and Pathogens in the Five Beekeeping Regions of Mexico

**DOI:** 10.3390/ani13111734

**Published:** 2023-05-24

**Authors:** Adriana Correa-Benítez, Ricardo Anguiano-Baez, Assad Heneidi-Zeckua, José L. Dávalos-Flores, Nelly T. Peña-Haaz, Eduardo E. Pérez-Martínez, Mariana Carbajal-Rodríguez, Itzel Vasquez-Valencia, Nayeli Almazán-Maldonado, Tatiana Petukhova, Ernesto Guzman-Novoa

**Affiliations:** 1 Departamento de Medicina y Zootecnia de Abejas, FMVZ, Universidad Nacional Autónoma de México, Cd. Universitaria, Mexico City 04510, Mexico; adrianac@unam.mx (A.C.-B.); ricardo.anguiano@comunidad.unam.mx (R.A.-B.); pehane@hotmail.com (N.T.P.-H.); m.carbajal.rodriguez@gmail.com (M.C.-R.); valitzel@yahoo.com.mx (I.V.-V.); nayalmazanm@gmail.com (N.A.-M.); 2 Consultoría en Epidemiología y Análisis de Riesgo, Adolfo Prieto 1543, Mexico City 03100, Mexico; aheneidi@gmail.com; 3 Departamento de Economía, Administración y Desarrollo Rural, FMVZ, Universidad Nacional Autónoma de México, Cd. Universitaria, Mexico City 04510, Mexico; jldf@unam.mx (J.L.D.-F.); eperez@tns.org (E.E.P.-M.); 4 Department of Population Medicine, University of Guelph, 50 Stone Road East, Guelph, ON N1G 2W1, Canada; tpetukho@uoguelph.ca; 5 School of Environmental Sciences, University of Guelph, 50 Stone Road East, Guelph, ON N1G 2W1, Canada

**Keywords:** *Apis mellifera*, honey bee diseases, *Varroa destructor*, *Nosema*, deformed wing virus, Israeli acute paralysis virus, sacbrood bee virus, small hive beetle, Mexico

## Abstract

**Simple Summary:**

Mexico is an important honey producer, but not much information exists about the health of honey bees in the country. This study analyzed the sanitary status of adult honey bees in five different beekeeping regions of Mexico. Samples from hives were analyzed to identify pests, parasites, and viruses. The mite *Varroa destructor* was found in 83.5% of the samples, with the Pacific Coast having the highest frequency (>95%) and levels (4.5% ± 0.6). Another mite, *Acarapis woodi*, was found in only one sample from the Pacific Coast, whereas the fungi *Nosema* spp. were present in 48.5% of the samples, with the highest frequency in the Yucatan Peninsula (64.6%). For viruses, deformed wing virus (DWV) was more frequently found in the Pacific Coast region (44.7%), and Israeli acute paralysis virus (IAPV) was detected in only 3.2% of the samples. Sacbrood bee virus (SBV) was frequently found in the High Plateau region (36.4%), and the small hive beetle (SHB) was common in the Yucatan Peninsula (39.2%). This information could be useful to design disease control strategies for honey bee colonies in different regions of Mexico.

**Abstract:**

Mexico is a major honey producer, but not much information exists about the health status of honey bees (*Apis mellifera* L.) in the country. This study was conducted to determine the sanitary status of adult honey bees in Mexico’s five beekeeping regions. Samples from 369 apiaries were diagnosed to identify pathogens such as *Varroa destructor*, which was quantified, *Acarapis woodi*, *Nosema* spp., and five viruses. Colonies were also inspected for the presence of the small hive beetle (SHB), *Aethina tumida*. *Varroa destructor* was found in 83.5% of the apiaries, with the Pacific Coast region having the highest prevalence (>95%) and rates (4.5% ± 0.6). *Acarapis woodi* was detected in only one apiary from the Pacific Coast, whereas *Nosema* spp. were prevalent in 48.5% of the apiaries, with the highest and lowest frequencies in the Yucatan Peninsula and North regions (64.6% and 10.2%, respectively). For viruses, deformed wing virus (DWV) was detected in 26.1% of the apiaries, with the highest frequency in the Pacific Coast region (44.7%). Israeli acute paralysis virus (IAPV) was diagnosed in 3.2% of the samples and sacbrood bee virus (SBV) in 23.3% of them, with the highest frequency in the High Plateau region (36.4%). Chronic bee paralysis and Kashmir bee viruses were not detected. SHB prevalence was 25.2% nationwide, with the highest frequency in the Yucatan Peninsula (39.2%). This study shows that the most common parasites of adult honey bees in Mexico are *V. destructor* and *Nosema* spp., and that the most prevalent virus is DWV, whereas SHB is highly prevalent in the Yucatan Peninsula. This information could be useful to design disease control strategies for honey bee colonies in different regions of Mexico.

## 1. Introduction

Mexico is an important beekeeping country, ranking among the top 10 nations with the most honey produced and exported in the world. There are nearly 2,000,000 managed honey bee (*Apis mellifera* L.) colonies in Mexico that produce over 60,000 tons of honey a year, of which more than half are exported [1]. Despite Mexico’s importance as a major honey producer, not much information exists about the health status of honey bees in the five beekeeping regions of the country. Isolated surveys have been carried out for specific pathogens at the local or state level, but none at the national level [2].

The ecto-parasitic mite *Varroa destructor* (Mesostigmata: Varroidae) is considered the pathogen that causes the most damage to the beekeeping industry worldwide [3,4,5]. The mite feeds upon the hemolymph and fat body of honey bees, inhibits their immune responses, shortens their lifespan, vectors viruses, and reduces colony honey production [4,6,7,8,9,10,11]. *Varroa destructor* was detected for the first time in Mexico in the state of Veracruz in 1992 [12] and is nowadays widely distributed throughout the country [2].

The honey bee tracheal mite (HBTM), *Acarapis woodi* (Acari: Tarsonemidae), is another serious parasite of honey bees that reproduces and spends most of its life cycle in the interior of the bees’ pro-thoracic tracheas, where it feeds on the host hemolymph by piercing tracheal tissue [13]. The mite shortens the honey bee lifespan and may reduce colony populations, honey production, and winter survival [14,15,16]. The HBTM was first discovered in Mexico in 1980 [17], and after that, it spread rapidly throughout the country [18].

Nosema disease, or nosemosis, is a parasitic disease of honey bees caused by two species of spore-forming microsporidian fungi, *Nosema apis* and *N. ceranae* (Dissociodihaplophasida: Nosematidae) [19]. These microsporidians infect and proliferate in the midgut epithelial cells of honey bees, causing digestive disorders that weaken them and shorten their life span, and may also reduce colony honey production [20,21]. *Nosema apis* has existed in Mexico for more than 60 years [22], but *N. ceranae* was first detected in samples from 2004 [23], although evidence of its earlier presence in the country was discovered later from older stored bee samples [24].

Regarding viruses that infect honey bees, a few of them may have a serious impact on their health. Among them, deformed wing virus (DWV), acute bee paralysis virus (ABPV), Israeli acute paralysis virus (IAPV), chronic bee paralysis virus (CBPV), and Kashmir bee virus (KBV) [25,26,27]. Another common virus found in brood and adult honey bees is the sacbrood bee virus (SBV), which does not seem to pose a major threat to honey bee health. In Mexico, the presence of DWV, ABPV, IAPV, KBV, and SBV has been reported and confirmed by molecular analyses, but only in some states or localities of the country [28,29,30].

The small hive beetle (SHB), *Aethina tumida* (Coleoptera: Nitidulidae), is a pest and scavenger of social bee colonies. The female lays eggs inside hives and the larvae feed upon bee brood, honey, and pollen, burrowing tunnels in the midrib of combs, which destroys them. The beetles also defecate in stored honey, spoiling it and sometimes causing the bees to abandon the hive [31]. The presence of SHB was first detected in Coahuila, Mexico (North region), in 2007 [32], and so far has been found in 15 of Mexico’s 32 states [33].

Several honey bee diseases and pests that are present in Mexico have been associated with massive colony losses worldwide [34,35,36]. In Mexico in particular, there have been recent cases of significant colony mortality in parts of the High Plateau region and in the north of the country, where losses ranged between 15% and 25% annually, from the years 2016 to 2019 [37]. The presence and distribution of pests and diseases in honey bee colonies in Mexico were recently reviewed [2]. The report highlights that the knowledge about the presence and distribution of honey bee pathogens in Mexico is incomplete and patchy, in part due to an insufficient number of surveys that have been conducted locally only, and because there are no databases on honey bee diseases at the national level.

The objective of this study was to conduct a survey of honey bee colonies at the national level within Mexico’s five beekeeping regions to determine their sanitary status for several diseases that affect adult bees, including varroosis (*V. destructor*), acarine disease (*A. woodi*), nosema disease (*Nosema* spp.), and five viral infections. Colonies were also inspected for the presence of SHB.

## 2. Materials and Methods

### 2.1. Beekeeping Regions and Sample Size

**Figure 1:**
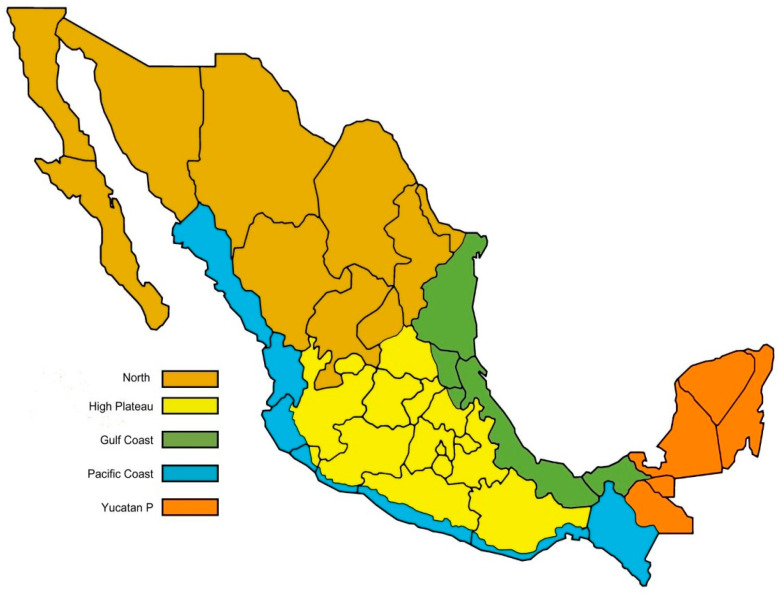
Map of Mexico showing the five beekeeping regions of the country.

A national-level survey was conducted to collect samples of adult honey bees from colonies located in the five beekeeping regions of Mexico (Figure 1). The number of samples collected was determined relative to the total number of apiaries in each region [38] to have a representative sample size. To estimate the sample size, variables such as estimated prevalence, sensitivity, specificity, population size, and confidence level were used with a probabilistic sampling design [39] using the *EpiTools epidemiological calculator*. A total of 369 sampling units (apiaries) under a stratified random sampling design was found to be a reliable sample size for this study. Because the majority of beekeepers in Mexico own between 25 and 210 colonies [38], small and sideline beekeeping operations were targeted for the survey. The numbers of samples collected per beekeeping region and state are shown in Table 1. In each apiary, one colony was randomly selected for sampling procedures, which took place during the summer months (between July and September 2017). All colonies sampled were queen right, had brood, and were kept in movable frame hives. Additionally, all apiaries sampled had been treated against varroa mite infestations with acaricides within the previous 12 months of the survey.

### 2.2. Collection of Samples

Each colony was sampled by opening the hive to first individually inspect every frame of the brood chamber on both sides, as well as the bottom board, for evidence of SHB. If visually detected, samples of beetles were collected in vials containing 70% ethanol for later confirmation [40]. Additionally, several samples of adult bees were collected as follows. (1) Approximately 300 adult bees were obtained from the brood nest of each colony and deposited in a 250 mL plastic jar containing 70% ethanol for diagnosis and quantification of *V. destructor* [41]. (2) Another sample of approximately 60–80 bees that were stored in ethanol as above was obtained from the hive entrance of each colony. This sample was used to diagnose HBTM and nosema disease (*Nosema* spp. spores) [42,43]. (3) An additional sample of 5 bees was obtained from the brood nest of each colony. The bees were handled with disposable gloves and introduced into sterile 5.0 mL microcentrifuge tubes containing RNAlater^®^ (Thermo Fisher Scientific; Mexico City, Mexico) for the preservation of viral RNA [44]. The samples for viral analyses were transported in a cooler with ice packs, and later stored at −70 °C until analyzed.

### 2.3. Laboratory Procedures

The samples were shipped to Centro Nacional de Servicios de Constatación en Salud Animal (CENAPA: Mexico’s Animal Health Diagnostic Centre) in Cuernavaca, Mor. (18.9° N, 99.2° W), where they were subjected to different diagnostic procedures as follows.

#### 2.3.1. Prevalence and Levels of Parasitic Mites

Adult bees and varroa mites were separated by agitating the jars containing the samples in ethanol as per De Jong et al. [45]. Mites and bees were counted to determine the percentage of mite infestation. For HBTM, 60 bees per sample (of those collected from the hive entrance) were dissected and microscopically analyzed as per Sammataro et al. [42] to detect *A. woodi* mites in the pro-thoracic tracheal tubes of the bees.

#### 2.3.2. Prevalence of *Nosema* spp.

For nosema disease, the abdomens of the same 60 bees used for HBTM diagnosis were macerated and three sub-samples from the macerate were mounted on slides to visually search for and identify *Nosema* spp. spores under a phase-contrast optic microscope as per Fries et al. [43].

#### 2.3.3. Viral Diagnosis

Samples were analyzed by RT-PCR to detect DWV, IAPV, CBPV, SBV, and KBV. Total RNA from three bees per sample was extracted with TRIzol^®^ (Thermo Fisher Scientific, Mexico City, Mexico) following the manufacturer’s instructions. Complementary DNA was obtained by reverse transcription using 2 µg of RNA with the RevertAid H Minus First Strand Synthesis Kit (Thermo Fisher Scientific) as per the manufacturer’s instructions.

The PCR reactions were performed with a Mastercycler (Eppendorf, Mexico City, Mexico). Each reaction contained 1.5 µL 10× PCR buffer (Thermo Fisher Scientific), 0.5 µL of 10 mM dNTPs (Bio Basic, Mexico City, Mexico), 0.2 µL of 5 U/µL Taq polymerase (Promega, Mexico City, Mexico), 2 µL of cDNA, 8.8 µL of ddH_2_O, and 1 µL of 10 µM forward and reverse primers of the corresponding virus. The primers used were those reported by Guzman-Novoa et al. [28]. The thermocycler was set to run for 3 min at 94 °C, then 35 cycles of 30 s each at 94 °C, followed by 1 min at 55 °C and 1 min at 72 °C, and a final extension at 72 °C for 10 min. These conditions were used for all viruses, except for DWV, the annealing temperature used was 58 °C. The PCR products were separated by electrophoresis on 1.1% agarose gels and stained with ethidium bromide. The amplified bands were photographed with a digital camera under a UV illuminator.

Two viruses, CBPV and KBV, were not detected in any of the samples. The only viruses detected by RT-PCR were DWV, IAPV, and SBV. PCR products of these viruses were purified and sequenced to confirm their identity (Genomic Sequencing Laboratory, Biology Institute, National University of Mexico). The sequences were blasted against viral sequences in the NCBI (GenBank acc. no. AJ489744.2, EF219380.1, and AF092924.1 for DWV, IAPV, and SBV, respectively). Nucleotide identity was >96% in all cases.

#### 2.3.4. Small Hive Beetle Diagnosis

Samples of collected beetles were morphologically diagnosed as per Neumann et al. [40], and the identity of *A. tumida* was confirmed in all cases.

### 2.4. Statistical Analyses

Descriptive statistical values were obtained, and the data were subjected to statistical tests. To determine if there were differences in the prevalence of pathogens or pests between regions, the data on positive and negative samples were analyzed with two-sample tests for equality of proportions using the Benjamini–Hochberg correction. For *V. destructor* infestation levels, the data were subjected to Shapiro–Wilk and Bartlett tests to analyze the assumptions of normality and homoscedasticity, respectively. The data did not have a normal distribution and therefore were analyzed with Kruskal–Wallis tests. All statistical analyses were carried out with R 3.3.1 (Foundation for Statistical Computing, Vienna, Austria).

## 3. Results

### 3.1. Parasitic Mites

**Figure 2:**
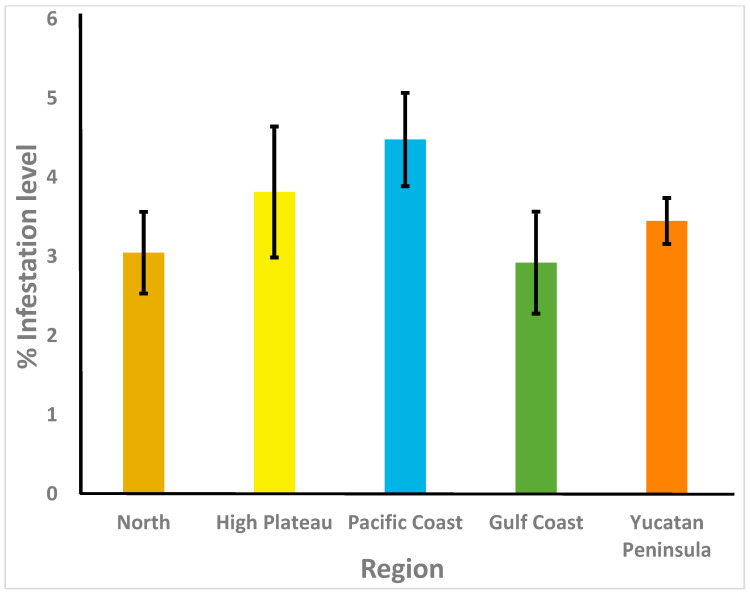
Varroa destructor infestation rates (% ± SE) in adult honey bees from colonies of Mexico’s five beekeeping regions. *n* = 369.

Results are shown in Table 2. *Varroa destructor* was detected in 83.5% of the apiaries sampled in the five beekeeping regions of Mexico, with the highest prevalence in the Pacific and Gulf Coast regions (95.7% and 95.0%, respectively), and the lowest prevalence in the High Plateau (79.5%) and the Yucatan Peninsula (79.9%) regions. In fact, the proportion of samples that were positive for *V. destructor* from these two regions was significantly lower than that of samples from the Pacific Coast region (*p* < 0.05) but did not differ from that of samples from the other two regions or from the national average (*p* > 0.05). The rates of *V. destructor* infestation varied between colonies from 0 to 34.5%, and the highest average levels were found in the Pacific Coast region (4.5 ± 0.6%), whereas the lowest average infestation rates were for the colonies sampled in the Gulf Coast region (2.9 ± 0.6%; Figure 2). However, there were no significant differences between regions for rates of *V. destructor* parasitism (H = 7.54, df = 4, *p* = 0.10). *Acarapis woodi* was found in only one apiary from the Pacific Coast region (2.1%), and there were no significant differences between regions for the prevalence of the parasite (*p* < 0.05).

### 3.2. Nosema spp.

Results are shown in Table 2. *Nosema* spp. spores were found in 48.5% of the sampled apiaries, with the highest and lowest frequencies in the Yucatan Peninsula and the North region (64.6% and 10.2%, respectively). *Nosema* spp. spores were significantly more prevalent in the Yucatan Peninsula than in the other four regions of the country (*p* < 0.05), whereas in the North region, *Nosema* spp. spores were significantly less prevalent than in the two coastal regions and the Yucatan Peninsula (*p* < 0.05) but did not differ in prevalence compared with samples from the High Plateau (*p* > 0.05).

### 3.3. Viruses

Results are shown in Table 3. DWV was detected in 26.1% of the apiaries nationwide, with the highest frequency in the Pacific and Gulf Coast regions (44.7% and 40.0%, respectively), and the lowest in the North region (16.7%), which differed significantly from the coastal regions for DWV prevalence (*p* < 0.05). IAPV was found in only 3.2% of the country’s samples and was not detected in the Pacific Coast region. No significant differences in IAPV prevalence were found between regions (*p* > 0.05). SBV was detected in 23.3% of the total samples, with a higher frequency in the High Plateau region (36.4%) compared to the other regions. However, significant differences between regions for SBV prevalence were only found between the High Plateau and the Gulf Coast region (*p* < 0.05).

### 3.4. Small Hive Beetle

**Figure 3:**
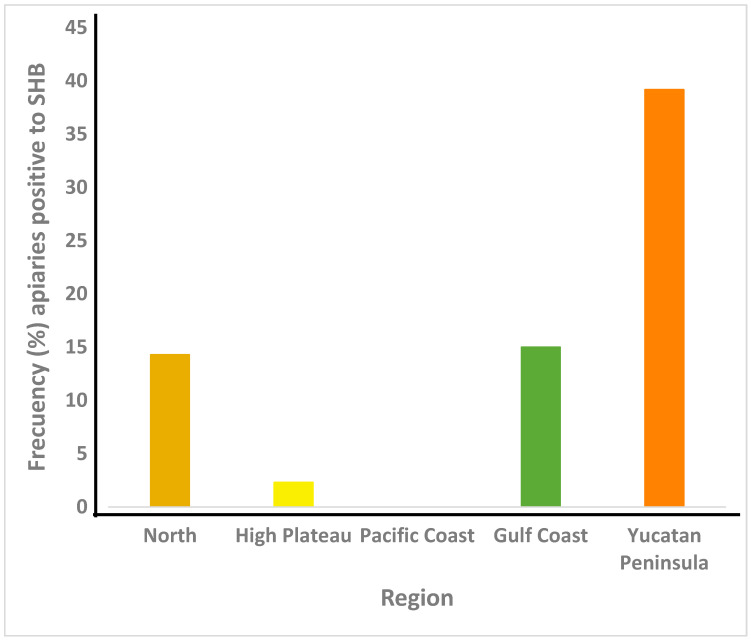
*Aethina tumida* prevalence (%) in honey bee colonies from apiaries of Mexico’s five beekeeping regions. *n* = 369.

The results are shown in Figure 3. *Aethina tumida*’s prevalence was 25.2% nationwide with significant differences between regions (*p* < 0.05). The highest frequency of SHB occurred in the Yucatan Peninsula (39.2%), and it was not found in the Pacific Coast region. Where SHB was detected, the lowest prevalence was for the High Plateau region (2.3%).

## 4. Discussion

This was the first survey conducted at the national level to determine the sanitary status of adult honey bees from the five different beekeeping regions of Mexico. The study was designed to sample the regions proportionally to the number of apiaries in each of them to have a representative sample of all five regions. The study focused on small and sideline operations because most beekeepers in Mexico belong to these categories [38]. Some pathogens were prevalent and abundant (*V. destructor*) in some regions, but not in others. It was evident that parasitic mites and DWV were more frequently found in the Pacific Coast region, whereas the frequency of *Nosema* spp. was significantly higher in the Yucatan Peninsula than in the North of Mexico. Additionally, SHB was significantly more prevalent in the Yucatan Peninsula than in the High Plateau region, whereas it was not detected in the Pacific Coast region. These associations and differences in the prevalence of particular pathogens and pests between regions are probably due to conditions in the regions that differentially favor their presence and abundance.

*Varroa destructor* was the parasite most frequently detected in the sampled apiaries. It was found in 83.5% of the samples at the national level. However, the frequency of *V. destructor*-positive apiaries was higher in the coastal regions (>95%) compared to the other beekeeping regions of Mexico. Previous studies conducted at the state or local levels have shown similar or slightly lower *V. destructor* prevalence in honey bee colonies. For example, in the southern area of Jalisco state, which is part of the Pacific Coast region, *V. destructor* was diagnosed in 88% of the sampled apiaries [46]. Another state-level survey conducted in Zacatecas also reported a frequency of 88% varroa mite-positive colonies [6]. Furthermore, a study conducted in the state of Yucatan reported a prevalence of less than 63% for *V. destructor* [47], which also coincides with the results of this study in that the frequency of *Varroa*-positive colonies of the Yucatan Peninsula was relatively lower than in most regions of Mexico. Nevertheless, overall, the prevalence of *V. destructor* in honey bee colonies of Mexico is high and possibly underestimated because the methods used are not 100% reliable. This is not surprising because the current beekeeping practices, as well as honey bee behavior, favor the dispersion of the varroa mite between colonies. Honey bees frequently drift to nearby colonies when returning from foraging trips, carrying mites with them [48]. Robbing behavior also favors mite dispersion [4]. Additionally, the relatively short distance between hives and apiaries contributes to varroa mite spread [49].

The mean rate of *Varroa* parasitism found in colonies of each beekeeping region was in general low (2.9–4.5%), and there were no significant differences between regions for mite infestation rates. These low mite infestation levels of honey bee colonies in Mexico may be the result of multiple factors, including different treatment strategies implemented in beekeeping operations, and the Africanization of honey bees. Beekeepers of all regions sampled reported having used acaricides to control varroa mite infestations in their colonies. Africanized bees (descendants of *A. mellifera scutellata*) have demonstrated in many studies to be more resistant to *V. destructor* than European honey bees [50,51,52,53,54], which could explain, at least partially, the low infestation rates of *V. destructor* found in this survey and in previous surveys conducted in several regions of Mexico. Honey bee colonies from all beekeeping regions of Mexico show some degree of Africanization based on morphometric and mt DNA analyses [52].

*Acarapis woodi* was diagnosed in only 0.3% of the colonies at the national level and was found in only one beekeeping region, although again, the method used to identify the mite may fail to detect it at very low levels of infestation. Similarly, other relatively recent surveys conducted in Mexico have failed to identify the mite, or have found it at a very low frequency (<1%) in surveys conducted in the High Plateau region [55,56]. Conversely, in surveys conducted between 1981 and 1983, 3 of every 10 colonies sampled in the five beekeeping regions of Mexico were positive for the mite [18,57]. In the decade of the 1980s, the HBTM was seen as responsible for the death of thousands of colonies in Mexico and the USA [14,57]. More recently, like in Mexico, *A. woodi* has been found at extremely low frequency and levels in the USA [58]. It seems that the most susceptible colonies were wiped out by HBTM infestations, and that natural selection favored the reproduction and survivorship of more resistant strains of honey bees, or perhaps that current methods of mite control are more effective. Alternatively, the mite could have evolved to be less virulent. The above potential explanations remain to be demonstrated with additional research. Nevertheless, beekeepers must remain vigilant of potential HBTM outbreaks in the future.

*Nosema* spp. spores were found in 48.5% of the apiaries at the country level, with prevalence ranging from 10.2% in the North to 64.6% in the Yucatan Peninsula. The frequency of this parasitism in the Yucatan Peninsula was significantly higher than in all other regions, suggesting that conditions in the peninsula favor the spread of the parasitosis between colonies. These results and conclusions are supported by previous studies at a local level that have reported a high prevalence of nosema disease in the state of Yucatan compared to other states of the country [47,59]. It is known that crowded hive conditions and humidity promote the spread and multiplication of *Nosema* microsporidians [19,20]. These conditions occur in the Yucatan Peninsula, which is very humid and has the highest concentration of colonies in Mexico [60]. Other humid and colony-crowded areas of Mexico, such as the coastal regions, followed the Yucatan Peninsula in the frequency of nosema disease. The least-colony-crowded and humid regions (North and High Plateau) had the lowest frequencies of the disease, although a recent study in the state of Baja California (Northwest of Mexico) diagnosed nosemosis by RT-PCR, finding a disease prevalence >50% [61]. In our study, the samples were not analyzed for *Nosema* species, something that should be considered in future surveys.

Regarding viruses, only three of them were detected: DWV, IAPV, and SBV. CBPV and KBV were not diagnosed in any of the samples. The two most frequently detected viruses at the national level were DWV and SBV (26.1% and 23.3%, respectively). For DWV, this is not surprising, since it seems to be the most prevalent virus in adult honey bees in many American countries [62,63,64,65]. The highest prevalence of DWV was found in the coastal regions, which also had the highest varroa mite prevalence. *Varroa destructor* is the main vector and driver of DWV infections in honey bee colonies because the virus reproduces in the salivary glands of the mite before being transmitted [66,67]. The North region had the lowest frequency of DWV, only 16.7%, which warrants further investigation.

A few previous studies had reported the presence of DWV in localities within the states of Chihuahua in the North, Nayarit in the Pacific Coast, and the Federal District in the High Plateau region [28,29,30,68], although its prevalence and intensity were not determined. More recently, a study conducted in the state of Jalisco reported the frequency and levels of *V. destructor* and DWV in honey bee colonies of African or European descent [69]. The investigators found that the prevalence and levels of both mite and DWV were significantly higher in colonies of European descent than in colonies of African descent that were in the Pacific Coast area of the state. The referred study also found a significant and positive correlation between mite infestation and DWV levels, and that the abundance of both pathogens was negatively correlated with the African ancestry of the colonies. The above results indicate that *V. destructor* influences the prevalence and levels of DWV and that the genotype of honey bee colonies influences their resistance to DWV. In our study, we also found a high prevalence of DWV in a region with a high mite prevalence, which, along with the results by Ramos-Cuellar et al. [69], strongly indicates that *V. destructor* plays an important role in the presence of DWV. We did not determine the level of Africanization of the bees sampled, something that warrants further investigation—although as previously mentioned, colonies in all beekeeping regions of Mexico are Africanized to some degree [52].

The second virus most frequently detected in this survey was SBV, which was particularly prevalent in the High Plateau region. The presence of SBV in Mexico has been reported since 1984 [70], then diagnosed by clinical symptoms. It was also detected by RT-PCR in 2012 in the High Plateau region [28]. This virus has been associated with inbreeding and is vertically transmitted by the queen [71]; thus, beekeepers are advised to requeen their colonies. Nevertheless, the reasons why SBV is more prevalent in the High Plateau than in other regions of Mexico require further research. In the case of IAPV, its frequency was low in all regions and thus does not seem to be a virus of importance in Mexico, but future surveys conducted during different seasons and years would be required to confirm these results.

*Aethina tumida* was found in all regions of Mexico, except in the Pacific Coast, and was particularly highly prevalent in the Yucatan Peninsula (39%). The prevalence of SHB, as determined in this study, may be sub-estimated since the diagnosis was performed visually by inspecting the combs and bottom boards of hives. The beetles are small and can easily hide under bees and debris. Therefore, it might be that we were not able to detect the presence of SHB in colonies of the Pacific Coast region. Nevertheless, the reasons for the relatively high frequency of SHB in honey bee colonies of the Yucatan Peninsula deserve attention. Therefore, additional research is recommended on the factors that may favor the presence and abundance of SHB, and whether its high prevalence is impacting the productivity of honey bee colonies in that region of Mexico. It is believed that the warm, humid conditions and the high density of managed and feral colonies of the Yucatan Peninsula favor the dispersion of SHB [33], although more research is needed to confirm this hypothesis. This beetle has been also found in colonies of stingless bees in Campeche state [72], part of the Yucatan Peninsula, which is worrisome because stingless bees are of great importance for honey production and folk medicine in rural Mayan communities of the region.

## 5. Conclusions

The beekeeping industry in Mexico is affected by parasitic and viral diseases, as well as by pests that impact the health and productivity of honey bee colonies. Therefore, it is important to generate knowledge on the presence and distribution of pathogens and pests in all regions of beekeeping importance in the country. This is the first survey conducted at the national level to determine the prevalence of the main pathogens and pests affecting adult honey bees in the five beekeeping regions of Mexico. The study showed that the most common parasites of adult honey bees in Mexico are *V. destructor* and *Nosema* spp., and that the most prevalent virus is DWV. SHB is particularly highly prevalent in the Yucatan Peninsula, which deserves further investigation because of the high beekeeping importance of that region. Additional surveys at different times of the year that include more pests and pathogens are needed to generate more accurate information on the health status of honey bees in Mexico, as well as to help international efforts to better document the distribution of honey bee diseases worldwide [73]. Studies on the actual impact that pathogens and pests may have on the health and productivity of honey bee colonies are also warranted. The information from such studies will help elucidate the dynamics of different diseases, as well as establish management strategies and policies of pathogen control to sustain healthy and productive colonies of honey bees so that they continue to have a positive impact on the economy of beekeepers and on managed and unmanaged ecosystems.

## Figures and Tables

**Table 1:** Regions, states, and number of apiaries of honey bees sampled in Mexico.

Region	State	No. Samples
North (*n* = 49)	Baja California Norte	5
	Baja California Sur	5
	Chihuahua	7
	Coahuila	5
	Durango	5
	Nuevo Leon	5
	San Luis Potosi	5
	Sonora	5
	Zacatecas	7
High Plateau (*n* = 44)	Aguascalientes	2
	México	5
	Guanajuato	6
	Guerrero	2
	Jalisco	4
	Michoacan	4
	Morelos	3
	Oaxaca	5
	Puebla	5
	Queretaro	1
	San Luis Potosi	3
	Tlaxcala	4
Pacific Coast (*n* = 47)	Chiapas	26
	Oaxaca	12
	Guerrero	3
	Colima	2
	Nayarit	2
	Sinaloa	2
Gulf Coast (*n* = 20)	Tamaulipas	4
	San Luis Potosi	2
	Hidalgo	1
	Queretaro	1
	Veracruz	11
	Tabasco	1
Yucatan Peninsula (*n* = 209)	Campeche	67
	Chiapas	10
	Quintana Roo	15
	Yucatan	117
Total		369

**Table 2:** Prevalence (%) of *Varroa destructor*, *Acarapis woodi*, and *Nosema* spp. in adult honey bees from apiaries of Mexico’s five beekeeping regions.

Region	*n*	*V. destructor* (%)	*A. woodi* (%)	*Nosema* spp. (%)
North	49	85.7 ^a,b^	0.0	10.2 ^a^
High Plateau	44	79.5 ^a^	0.0	22.7 ^a,b^
Pacific Coast	47	95.7 ^b^	2.1	44.7 ^c^
Gulf Coast	20	95.0 ^a,b^	0.0	40.0 ^b,c^
Yucatan Peninsula	209	79.9 ^a^	0.0	64.6 ^d^
National	369	83.5	0.3	48.5

Different superscript letters indicate significant differences (*p* < 0.05) between regions based on pairwise comparison tests on proportions with Benjamini–Hochberg probability correction. No differences were found for *A*. *woodi*.

**Table 3:** Prevalence (%) of deformed wing virus (DWV), Israeli acute paralysis virus (IAPV), and sacbrood bee virus (SBV) in adult honey bees from apiaries of Mexico’s five beekeeping regions.

Region	*n*	DWV (%)	IAPV (%)	SBV (%)
North	49	16.7 ^a^	10.2	18.4 ^a,b^
High Plateau	44	25.0 ^a,b^	2.3	36.4 ^b^
Pacific Coast	47	44.7 ^b^	0.0	21.3 ^a,b^
Gulf Coast	20	40.0 ^b^	5.0	10.0 ^a^
Yucatan Peninsula	209	23.4 ^a,b^	2.4	23.4 ^a,b^
National	369	26.1	3.2	23.3

Different superscript letters indicate significant differences (*p* < 0.05) between regions based on pairwise comparison tests on proportions with Benjamini–Hochberg probability correction.

## Data Availability

The data of this study will be made available from the corresponding author upon reasonable request.
